# Cytotoxic T Lymphocyte-Associated Antigen 4 Gene Polymorphisms and Autoimmune Thyroid Diseases: An Updated Systematic Review and Cumulative Meta-Analysis

**DOI:** 10.1155/2015/747816

**Published:** 2015-03-24

**Authors:** Hai-Feng Hou, Xu Jin, Tao Sun, Cheng Li, Bao-Fa Jiang, Qun-Wei Li

**Affiliations:** ^1^School of Public Health, Taishan Medical University, Taian 271000, China; ^2^School of Basic Medical Sciences, Taishan Medical University, Taian 271000, China; ^3^Ruijin Hospital, Shanghai Jiao Tong University, Shanghai 200025, China; ^4^School of Public Health, Shandong University, Jinan 250012, China

## Abstract

The association of the cytotoxic T lymphocyte-associated antigen 4 (CTLA-4) gene and susceptibility to autoimmune thyroid diseases (AITDs) has been studied extensively. However, the results were not the same in different ethnic groups. We updated the meta-analysis of association of CTLA-4 gene polymorphisms with AITDs and summarized the results in specific ethnicity. The associations of A49G gene polymorphism with GD, A49G gene polymorphism with HT, CT60 gene polymorphism with GD, and CT60 gene polymorphism with HT were summarized based on the literatures published up to October 30, 2014, in English or Chinese languages. The participants involved in the studies of A49G with GD, A49G with HT, CT60 with GD, and CT60HT were 39004 subjects (in 51 studies), 13102 subjects (in 22 studies), 31446 subjects (in 22 studies), and 6948 subjects (in 8 studies), respectively. The pooled ORs of CTLA-4 gene polymorphisms with AITDs were larger than 1.00, and the 95% CIs of ORs were statistically significant among whole population analyses. However, the subgroup analysis demonstrated that pooled ORs of A49G polymorphisms with GD among Africans or Americans are less than 1.00. The accumulated evidence suggests that the G allele mutant of A49G and CT60 increased the risks of HT and GD.

## 1. Introduction

Autoimmune thyroid diseases (AITDs) are the most popular autoimmune thyroid diseases; hyperthyroid Graves' disease (GD) and Hashimoto's (goitrous) thyroiditis (HT) are two common types of AITDs. It is well known that AITDs are caused partly by specific genetic background [1]. The association of the cytotoxic T lymphocyte-associated antigen 4 (CTLA-4) gene and susceptibility to AITDs has been studied extensively [2–4]. The CTLA-4 gene is located on the region of human chromosome 2q33 and encodes the immunoregulatory molecule. It is proved to be a key negative regulator of T-cell activity [5, 6]. Single nucleotide polymorphisms (SNPs) at position 49 in exon 1 (+49 A/G, A49G, rs231775) and +6230 G/A (CT60, rs3087243) showed an association with AITDs. A comprehensive meta-analysis including 43 studies and more than 13,000 subjects was published in 2007 [7]. Subsequently, about 30 studies that investigated the relationship between the CTLA-4 gene SNPs and AITDs have been published. We designed the current systematic review and cumulative meta-analysis to include the most recent data and summarized the results with more genetic models.

## 2. Methods

### 2.1. Identification of Eligible Studies

The literature published up to October 30, 2014, in English or Chinese was searched in the MEDLINE, EMBASE, and China Biology Medicine disc (CBMdisc) databases. The search strategy was based on the key terms of “CTLA4,” “CTLA-4,” “cytotoxic T-cell lymphocyte associated antigen 4,” “CD28,” “CD152,” “Graves' disease,” “GD,” “Hashimoto's thyroiditis,” and “HT.” Reference lists of relevant papers were reviewed to find additional studies. H.-F. Hou and X. Jin independently reviewed all studies and assessed the quality of each study according to the following inclusion criteria. (1) The publication was case-control study design, and the associations between A49G or CT60 genetic polymorphisms and AITDs were investigated. (2) Genotype distribution data were offered in both cases and controls. (3) For the overlapping data or the same papers, the largest population or the most recent study was included. (4) We limited the data to studies published in English and Chinese language. We compared our collection information with the data of Kavvoura et al. [7] on The Endocrine Society's Journals Online website (available at http://press.endocrine.org/journal/jcem) and adopted the unpublished studies provided in Kavvoura's meta-analysis.

### 2.2. Data Extraction

For published studies, two reviewers (H.-F. Hou and T. Sun) independently extracted data and resolved disagreements by discussion or with a third party (Li QW) when necessary. We collected the following information carefully: author name, journal source, publication year, ethnicity of study population (Asian, Caucasian, African, and American), the number of individuals in case and control groups, and genotype distribution of cases and controls.

### 2.3. Meta-Analysis Methods

The analysis of data was performed with Review Manager 5.3 (The Cochrane Collaboration, Oxford, UK). Allele frequencies at the A49G or CT60 gene polymorphisms from the literatures were calculated by the allele counting method. Four genetic models, (1) allele contrast (G versus A), (2) additive genetic model (GG versus AA), (3) dominant model (GG + AG versus AA), and (4) recessive model (GG versus AG + AA), were measured in this meta-analysis, and association values of the CTLA-4 genetic polymorphisms with risk of AITDs were estimated by odds ratios (ORs) and 95% confidence intervals (CIs). We also assessed Hardy-Weinberg Equilibrium (HWE) of genotype frequencies in the control group with a chi-square test, and *P* value < 0.05 was considered to be significant. The heterogeneity across all studies was tested by the *I* ^2^ statistics and chi-square-based *Q*-test. The heterogeneity was considered to be significantly large when *P* < 0.10 and *I* ^2^ > 50%. Then random effects model was used to combine eligible data. The statistical significance of pooled ORs was measured by the *Z*-test. Subgroup meta-analyses were conducted according to different ethnicities. In addition, sensitivity analysis was implemented to assess stability of the summary result by sequential removal of individual studies. Furthermore, publication bias was measured by funnel plots.

## 3. Results

### 3.1. Identification of Eligible Studies

Besides the 43 studies mentioned in Kavvoura et al.'s meta-analysis [7], 25 additional studies were included in this review (Table 1). Sixteen studies were English language publications [8–23] and 9 studies were published in Chinese [24–32]. Thus, the present updated meta-analysis consisted of 68 studies.

### 3.2. Quantitative Analysis

#### 3.2.1. A49G Gene Polymorphism and GD

**Figure 1:**
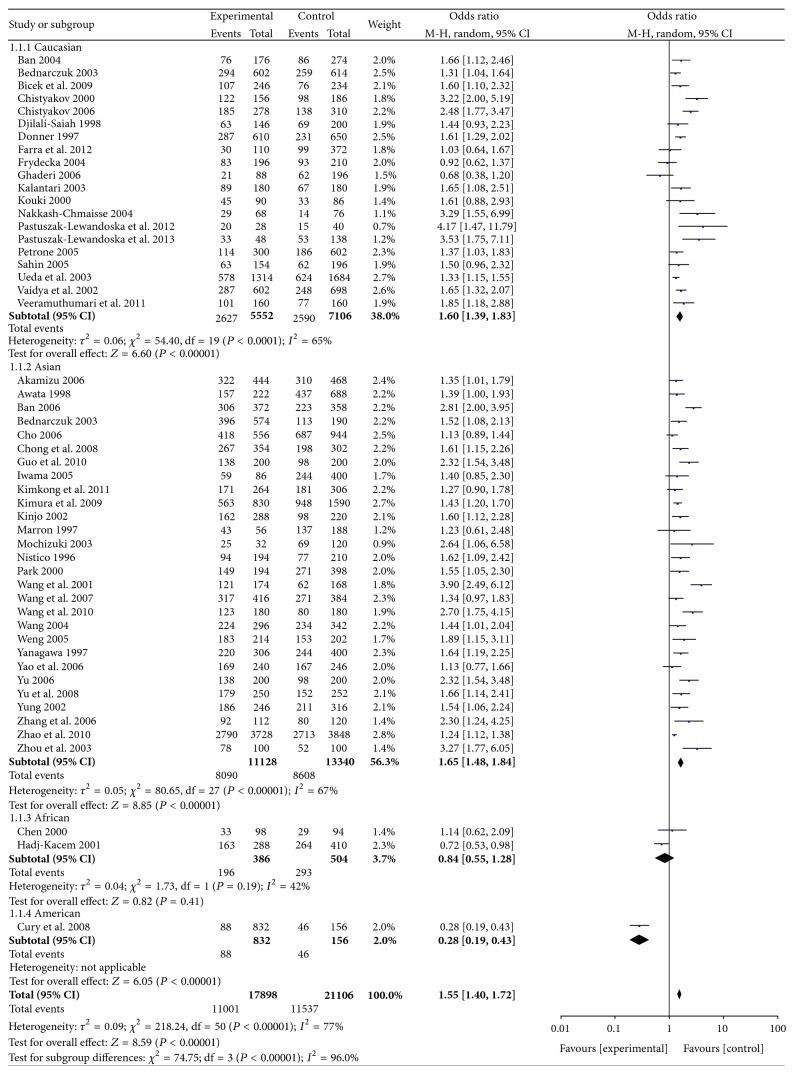
Forest plot of the association between an allele model of A49G polymorphism and GD.

**Figure 2:**
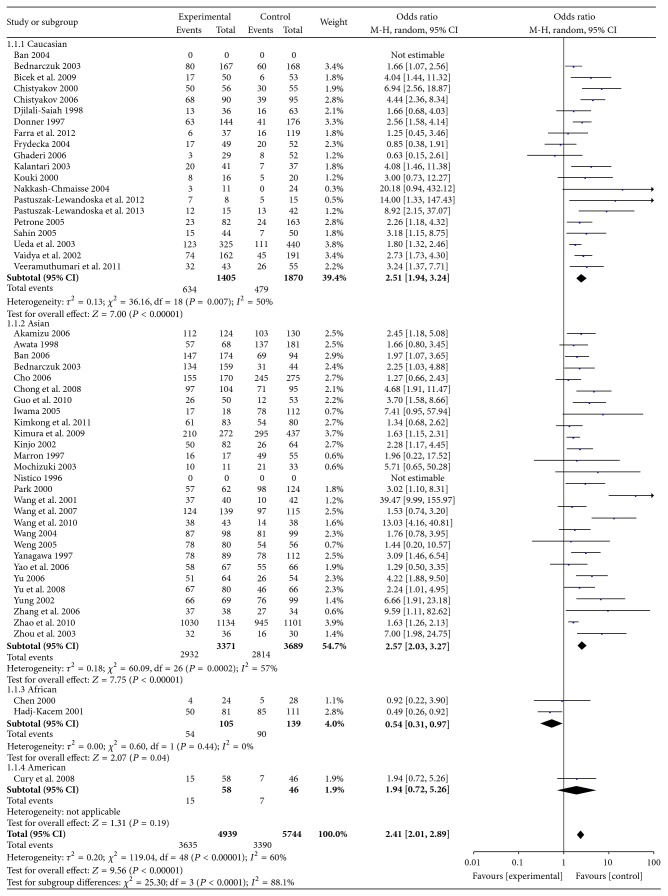
Forest plot of the association between an additive model of A49G polymorphism and GD.

The summary OR of included studies was increased 1.55-fold in susceptibility to GD in subjects with the G allele, and the associations of GD and A49G polymorphisms were statistically significant in an additive genetic model (GG versus AA: OR = 2.41, 95% CI: 2.01–2.89), a dominant genetic model (GG + AG versus AA: OR = 1.76, 95% CI: 1.52–2.03), and a recessive genetic model (GG versus AG + AA: OR = 1.79, 95% CI: 1.58–2.02). The detailed results were shown in Figures 1 and 2 and Supplemental Figures 1 and 2 in Supplementary Material available online at http://dx.doi.org/10.1155/2015/747816.

The subgroup analysis was performed by ethnicity to decrease the heterogeneity. As shown in Figures 1 and 2, significant associations between A49G SNP and GD risk were identified in Asians and Caucasians.

#### 3.2.2. A49G Gene Polymorphism and HT

**Figure 3:**
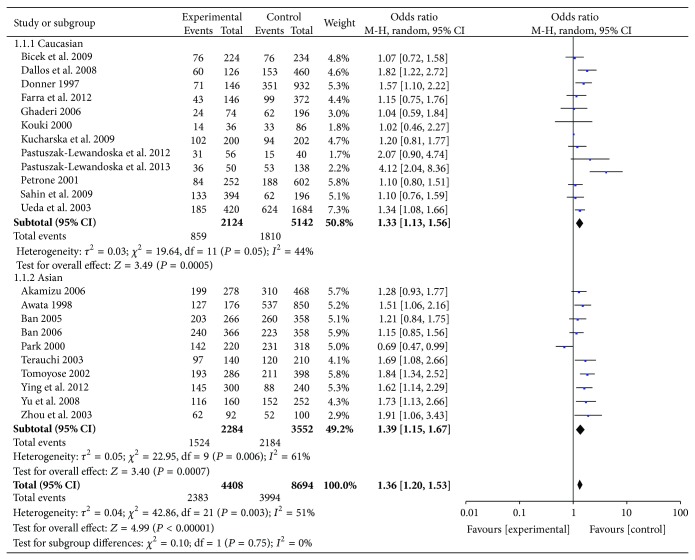
Forest plot of the association between an allele model of A49G polymorphism and HT.

The meta-analysis suggested (see Figure 3 and Supplemental Figures 3–5) that A49G polymorphisms increased the risk of HT significantly in the allele frequencies (G versus A: OR = 1.36, 95% CI: 1.20–1.53), the additive genotype (GG versus AA: OR = 2.10, 95% CI: 1.75–2.51), the dominant genotype (GG + AG versus AA: OR = 1.57, 95% CI: 1.26–1.96), and the recessive genotype (GG versus AG + AA: OR = 1.46, 95% CI: 1.19–1.81). The subgroup analyses showed that A49G polymorphism was one of the risk factors for GD in Asians and Caucasians.

#### 3.2.3. CT60 Gene Polymorphism and GD

**Figure 4:**
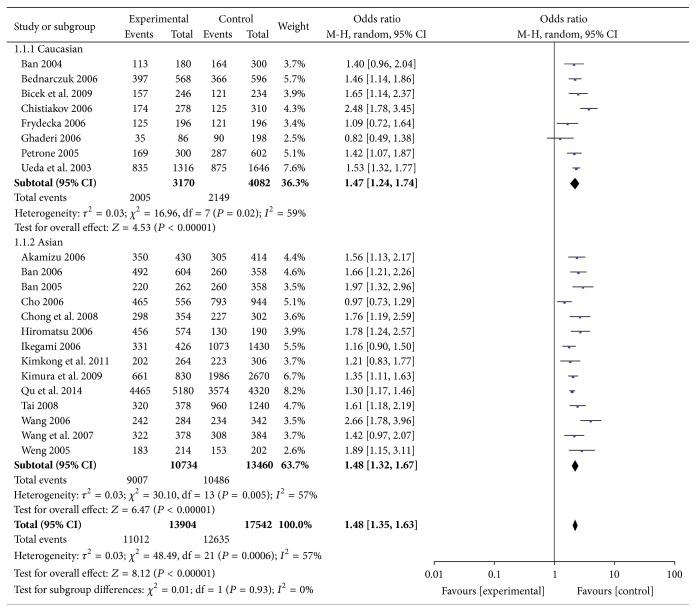
Forest plot of the association between an allele model of CT60 polymorphism and GD.

The summary analyses of CT60 gene polymorphism and GD are shown in Figure 4 and Supplemental Figures 6–8. The pooled ORs of CT60 polymorphisms with GD in allele frequencies, the additive genetic model, the dominant genetic model, and the recessive genetic model were 1.48 (95% CI: 1.35–1.63), 1.98 (95% CI: 1.73–2.27), 1.72 (95% CI: 1.52–1.96), and 1.56 (95% CI: 1.39–1.76), respectively. The subgroup analyses suggested that CT60 polymorphism was a risk factor for GD in Asians and Caucasians.

#### 3.2.4. CT60 Gene Polymorphism and HT

**Figure 5:**
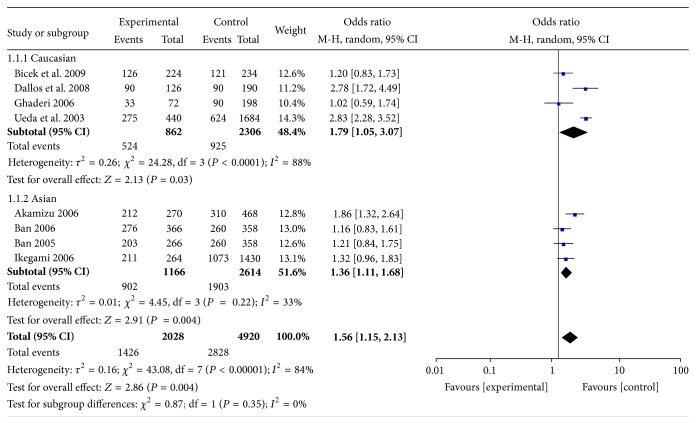
Forest plot of the association between an allele model of CT60 polymorphism and HT.

As shown in Figure 5 and Supplemental Figures 9–11, CT60 genetic polymorphisms increased HT risk significantly in the allele frequencies contrast (G versus A: OR = 1.56, 95% CI: 1.15–2.13), the additive genetic contrast (GG versus AA: OR = 2.58, 95% CI: 1.33–5.01), the dominant genetic contrast (GG + AG versus AA: OR = 1.95, 95% CI: 1.20–3.15), and the recessive genetic contrast (GG versus AG + AA: OR = 1.79, 95% CI: 1.20–2.67). The subgroup analyses showed that CT60 genetic polymorphism was one of the risk factors for GD in Asians and Caucasians.

### 3.3. Publication Bias

In order to evaluate publication bias in this updated systematic review, Begg's Funnel plots were performed, and the results showed that no obvious asymmetry existed for the meta-analyses of A49G and CT60 genetic polymorphisms.

### 3.4. Sensitivity Analysis

In order to conduct sensitivity analyses, we calculated the pooled ORs through removing each study sequentially and leaving out certain studies, such as studies conducted among special population. The analyses showed that the results were not changed significantly. However, the summary results of the association between CT60 and HT among Caucasians were shifted in the sensitivity analyses.

## 4. Discussion

GD and HT are the most prevalent autoimmune thyroid diseases (AITDs), which represent two opposite pathogenic paths: hyperthyroidism in GD and thyroid destruction in HT [15, 19]. Although the etiological mechanisms of GD and HT are not distinctly clarified, CTLA-4 gene polymorphisms (A49G and CT60) have been identified as the most important genetic factors in many genetic researches and genome-wide association study (GWAS) [2, 12]. A large-scale meta-analysis including 43 studies and more than 13,000 subjects was published in the present journal in 2007 [7]. The results identified the roles of A49G and CT60 gene polymorphism in AITDs. Subsequently, more than 30 studies repeatedly confirmed the associations of the CTLA-4 gene with GD and HT. The current updated meta-analysis included the most recent eligible studied and summarized the data in specific ethnicity.

A49G gene polymorphism was widely investigated for the susceptibility to AITDs; the G allelic gene variation was considered as a risk factor of GD and HT. Our current meta-analysis showed that A49G polymorphisms significantly increased the risk of GD in total population. Nevertheless, the genetic variation had a protective effect in Africans according to the additive model analysis. Furthermore, a total of 22 studies were summarized for the A49G gene polymorphism with HT. The results suggested that the polymorphism distinctly increases the risk of HT among Caucasians and Asians.

The G allele of CT60 gene is another focused genetic pathogenesis associated with HT and GD. A total of 22 studies were included in our meta-analysis for CT60 polymorphism and GD, and the pooled OR values indicated that G allele carriers might increase GD risk. Moreover, the summarized result involving 8 original studies suggested that CT60 polymorphisms were associated with susceptibility to HT among Caucasian and Asian population, except that no significant pooled OR was found in dominant genetic model of Caucasians.

In this updated meta-analysis, we guaranteed the stability of results with sensitivity analysis. No obvious publication bias existed according to funnel plot test. We performed heterogeneity test to assess the reliability of the results and conducted subgroup analysis.

There are some limitations in our study. The sample size in Africans or Americans was not large enough. More well-designed studies need to be conducted in Africans or Americans to clarify the associations of the CTLA-4 gene with AITDs.

## Supplementary Material

The online Supplementary Materials consist of the forest plots of the meta-analyses which were not provided in the published article. The associations of A49G polymorphism with GD in the dominant model and recessive model were shown in Supplemental Figures 1 and 2. The associations of A49G polymorphism with HT in the additive model, dominant model, and recessive model were shown in Supplemental Figures 3, 4, and 5. The associations of CT60 polymorphism with GD in the additive model, dominant model, and recessive model were shown in Supplemental Figures 6, 7, and 8. The associations of CT60 polymorphism with HT in the additive model, dominant model, and recessive model were shown in Supplemental Figures 9, 10, and 11, respectively.

## Figures and Tables

**Table 1:** Characteristics of new studies included in the meta-analysis.

Study	Year	Country	Ethnicity	Gene	Disease	Cases			Controls		
						AA	AG	GG	AA	AG	GG
Wang et al. [24]	2001	China	Asian	A49G	GD	37	47	3	27	26	7
Zhou et al. [25]	2003	China	Asian	A49G	GD	32	14	4	5	5	10
Zhang et al. [8]	2006	China	Asian	A49G	GD	37	18	1	26	25	29
Yao et al. [26]	2006	China	Asian	A49G	GD	58	53	9	55	57	11
Yu et al. [32]	2006	China	Asian	A49G	GD	51	36	13	26	46	28
Wang et al. [9]	2007	China	Asian	A49G	GD	124	69	15	46	60	20
Yu et al. [27]	2008	China	Asian	A49G	GD	67	45	13	13	27	29
Chong et al. [10]	2008	China	Asian	A49G	GD	97	73	7	16	67	103
Cury et al. [11]	2008	Brazil	American	A49G	GD	15	58	43	6	64	47
Bicek et al. [12]	2009	Slovenia	Caucasian	A49G	GD	17	73	33	14	52	24
Kimura et al. [13]	2009	Japan	Asian	A49G	GD	210	143	62	10	42	32
Wang et al. [28]	2010	China	Asian	A49G	GD	38	47	5	16	20	14
Guo et al. [29]	2010	China	Asian	A49G	GD	26	52	24	12	47	41
Zhao et al. [14]	2010	China	Asian	A49G	GD	1030	730	104	295	358	142
Pastuszak-Lewandoska et al. [15]	2012	Poland	Caucasian	A49G	GD	7	6	1	97	77	18
Veeramuthumari et al. [16]	2011	India	Caucasian	A49G	GD	32	37	11	71	56	24
Kimkong et al. [17]	2011	Thailand	Asian	A49G	GD	61	49	22	54	73	26
Farra et al. [18]	2012	Lebanon	Caucasian	A49G	GD	6	18	31	7	32	39
Pastuszak-Lewandoska et al. [19]	2013	Poland	Caucasian	A49G	GD	12	9	3	945	823	156

Pastuszak-Lewandoska et al. [15]	2012	Poland	Caucasian	A49G	HT	6	19	3	5	5	10
Zhou et al. [25]	2003	China	Asian	A49G	HT	24	14	8	46	60	20
Yu et al. [27]	2008	China	Asian	A49G	HT	41	34	5	15	64	22
Dallos et al. [20]	2008	Slovakia	Caucasian	A49G	HT	13	34	16	13	27	29
Kucharska et al. [21]	2009	Poland	Caucasian	A49G	HT	31	40	29	16	67	103
Bicek et al. [12]	2009	Slovenia	Caucasian	A49G	HT	15	46	51	6	64	47
Sahin et al. [22]	2009	Turk	Caucasian	A49G	HT	21	91	85	17	54	49
Farra et al. [18]	2012	Lebanon	Caucasian	A49G	HT	6	31	36	16	20	14
Ying et al. [30]	2012	China	Asian	A49G	HT	46	53	51	31	91	108
Pastuszak-Lewandoska et al. [19]	2013	Poland	Caucasian	A49G	HT	14	8	3	7	48	43

Wang et al. [9]	2007	China	Asian	CT60	GD	138	46	5	30	61	26
Chong et al. [10]	2008	China	Asian	CT60	GD	125	48	4	735	516	84
Tsai et al. [23]	2008	China	Asian	CT60	GD	136	48	5	125	58	9
Bicek et al. [12]	2009	Slovenia	Caucasian	CT60	GD	50	57	16	88	51	12
Kimura et al. [13]	2009	Japan	Asian	CT60	GD	267	127	21	82	59	12
Kimkong et al. [17]	2011	Thailand	Asian	CT60	GD	78	46	8	372	216	32
Qu et al. [31]	2014	China	Asian	CT60	GD	1989	487	114	1550	474	136

Dallos et al. [20]	2008	Slovakia	Caucasian	CT60	HT	31	28	4	20	50	25
Bicek et al. [12]	2009	Slovenia	Caucasian	CT60	HT	37	52	23	30	61	26
