# Supplementary material for: Cytotoxic T Lymphocyte-Associated Antigen 4 Gene Polymorphisms and Autoimmune Thyroid Diseases: An Updated Systematic Review and Cumulative Meta-Analysis
Source: Int J Endocrinol. 2015 Mar 24;2015:747816. doi: 10.1155/2015/747816 (PMC4387902; doi:10.1155/2015/747816)
Supplement: Supplementary file 1 — The online Supplementary Materials consist of the forest plots of the meta-analyses which were not provided in the published article. The associations of A49G polymorphism with GD in the dominant model and recessive model were shown in Supplemental Figures 1 and 2. The associations of A49G polymorphism with HT in the additive model, dominant model, and recessive model were shown in Supplemental Figures 3, 4, and 5. The associations of CT60 polymorphism with GD in the additive model, dominant model, and recessive model were shown in Supplemental Figures 6, 7, and 8. The associations of CT60 polymorphism with HT in the additive model, dominant model, and recessive model were shown in Supplemental Figures 9, 10, and 11, respectively. [file 747816.f1.pdf]

Supplemental Figures

Study or Subgroup	Experimental Events	Experimental Total	Control Events	Control Total	Weight	Odds Ratio M-H, Random, 95% CI
**1.1.1 Caucasian**						
Ban2004	0	0	0	0		Not estimable
Bednarczuk2003	214	301	199	307	3.5%	1.33 [0.95, 1.88]
Bicek2009	90	123	70	117	2.7%	1.83 [1.06, 3.15]
Chistyakov2000	72	78	68	93	1.5%	4.41 [1.71, 11.42]
Chistyakov2006	117	139	99	155	2.6%	3.01 [1.72, 5.27]
Djilali-Saiah1998	50	73	53	100	2.4%	1.93 [1.03, 3.62]
Donner1997	224	305	190	325	3.5%	1.96 [1.40, 2.75]
Farra2012	24	55	83	186	2.5%	0.96 [0.52, 1.76]
Frydecka2004	66	98	73	105	2.5%	0.90 [0.50, 1.63]
Ghaderi2006	18	44	54	98	2.1%	0.56 [0.27, 1.16]
Kalantari2003	69	90	60	90	2.3%	1.64 [0.85, 3.17]
Kouki2000	37	45	28	43	1.4%	2.48 [0.92, 6.66]
Nakkash-Chmaisse2004	26	34	14	38	1.4%	5.57 [1.99, 15.62]
Pastuszak-Lewandoska2012	13	14	10	20	0.4%	13.00 [1.42, 119.07]
Pastuszak-Lewandoska2013	21	24	40	69	1.0%	5.08 [1.38, 18.64]
Petrone2005	91	150	162	301	3.3%	1.32 [0.89, 1.97]
Sahin2005	48	77	55	98	2.5%	1.29 [0.70, 2.38]
Ueda2003	455	657	513	842	4.0%	1.44 [1.16, 1.79]
Vaidya2003	213	301	203	349	3.6%	1.74 [1.26, 2.41]
Veeramuthumari2011	69	80	51	80	1.9%	3.57 [1.63, 7.80]
**Subtotal (95% CI)**		**2688**		**3416**	**45.1%**	**1.73 [1.41, 2.11]**
Total events	1917		2025			
Heterogeneity: Tau² = 0.10; Chi² = 46.01, df = 18 (P = 0.0003); I² = 61%						
Test for overall effect: Z = 5.28 (P < 0.00001)						
**1.1.2 Asian**						
Akamizu2006	210	222	207	234	2.1%	2.28 [1.13, 4.63]
Awata1998	100	111	300	344	2.2%	1.33 [0.66, 2.68]
Ban2006	159	186	154	179	2.5%	0.96 [0.53, 1.72]
Bednarczuk2003	262	287	82	95	2.1%	1.66 [0.81, 3.40]
Cho2006	263	278	442	472	2.4%	1.19 [0.63, 2.25]
Chong2008	170	177	127	151	1.7%	4.59 [1.92, 10.98]
Guo2010	78	100	72	100	2.3%	1.38 [0.72, 2.62]
Iwama2005	42	43	166	200	0.5%	8.60 [1.14, 64.67]
Kimkong2011	110	132	127	153	2.4%	1.02 [0.55, 1.91]
Kimura2009	353	415	653	795	3.6%	1.24 [0.89, 1.71]
Kinjo2002	112	144	72	110	2.7%	1.85 [1.06, 3.22]
Marron1997	27	28	88	94	0.4%	1.84 [0.21, 15.97]
Mochizuki2003	15	16	48	60	0.4%	3.75 [0.45, 31.27]
Nistico1996	0	0	0	0		Not estimable
Park2000	92	97	173	199	1.4%	2.77 [1.03, 7.44]
Wang-L2001	84	87	52	84	1.1%	17.23 [5.02, 59.12]
Wang-PW2007	193	208	174	192	2.1%	1.33 [0.65, 2.72]
Wang-SQ2010	85	90	66	90	1.4%	6.18 [2.24, 17.07]
Wang2004	137	148	153	171	1.9%	1.47 [0.67, 3.21]
Weng2005	105	107	99	101	0.5%	1.06 [0.15, 7.67]
Yanagawa1997	142	153	166	200	2.1%	2.64 [1.29, 5.41]
Yao2006	111	120	112	123	1.6%	1.21 [0.48, 3.04]
Yu2006	87	100	72	100	2.1%	2.60 [1.26, 5.39]
Yu2008	112	125	106	126	2.0%	1.63 [0.77, 3.43]
Yung2002	120	123	135	158	1.1%	6.81 [2.00, 23.27]
Zhang2006	55	56	53	60	0.4%	7.26 [0.86, 61.06]
Zhao2010	1760	1864	1768	1924	3.8%	1.49 [1.16, 1.93]
Zhou2003	46	50	36	50	1.1%	4.47 [1.36, 14.76]
**Subtotal (95% CI)**		**5467**		**6565**	**47.9%**	**1.93 [1.55, 2.40]**
Total events	5030		5703			
Heterogeneity: Tau² = 0.14; Chi² = 56.28, df = 26 (P = 0.0005); I² = 54%						
Test for overall effect: Z = 5.94 (P < 0.00001)						
**1.1.3 African**						
Chen2000	29	49	24	47	1.9%	1.39 [0.62, 3.12]
Hadj-Kacem2001	113	144	179	205	2.6%	0.53 [0.30, 0.94]
**Subtotal (95% CI)**		**193**		**252**	**4.5%**	**0.82 [0.32, 2.11]**
Total events	142		203			
Heterogeneity: Tau² = 0.34; Chi² = 3.65, df = 1 (P = 0.06); I² = 73%						
Test for overall effect: Z = 0.41 (P = 0.68)						
**1.1.4 American**						
Cury2008	73	116	39	78	2.6%	1.70 [0.95, 3.04]
**Subtotal (95% CI)**		**116**		**78**	**2.6%**	**1.70 [0.95, 3.04]**
Total events	73		39			
Heterogeneity: Not applicable						
Test for overall effect: Z = 1.78 (P = 0.07)						
**Total (95% CI)**		**8464**		**10311**	**100.0%**	**1.76 [1.52, 2.03]**
Total events	7162		7970			
Heterogeneity: Tau² = 0.13; Chi² = 116.83, df = 48 (P < 0.00001); I² = 59%						
Test for overall effect: Z = 7.62 (P < 0.00001)						
Test for subgroup differences: Chi² = 3.25, df = 3 (P = 0.35), I² = 7.6%						

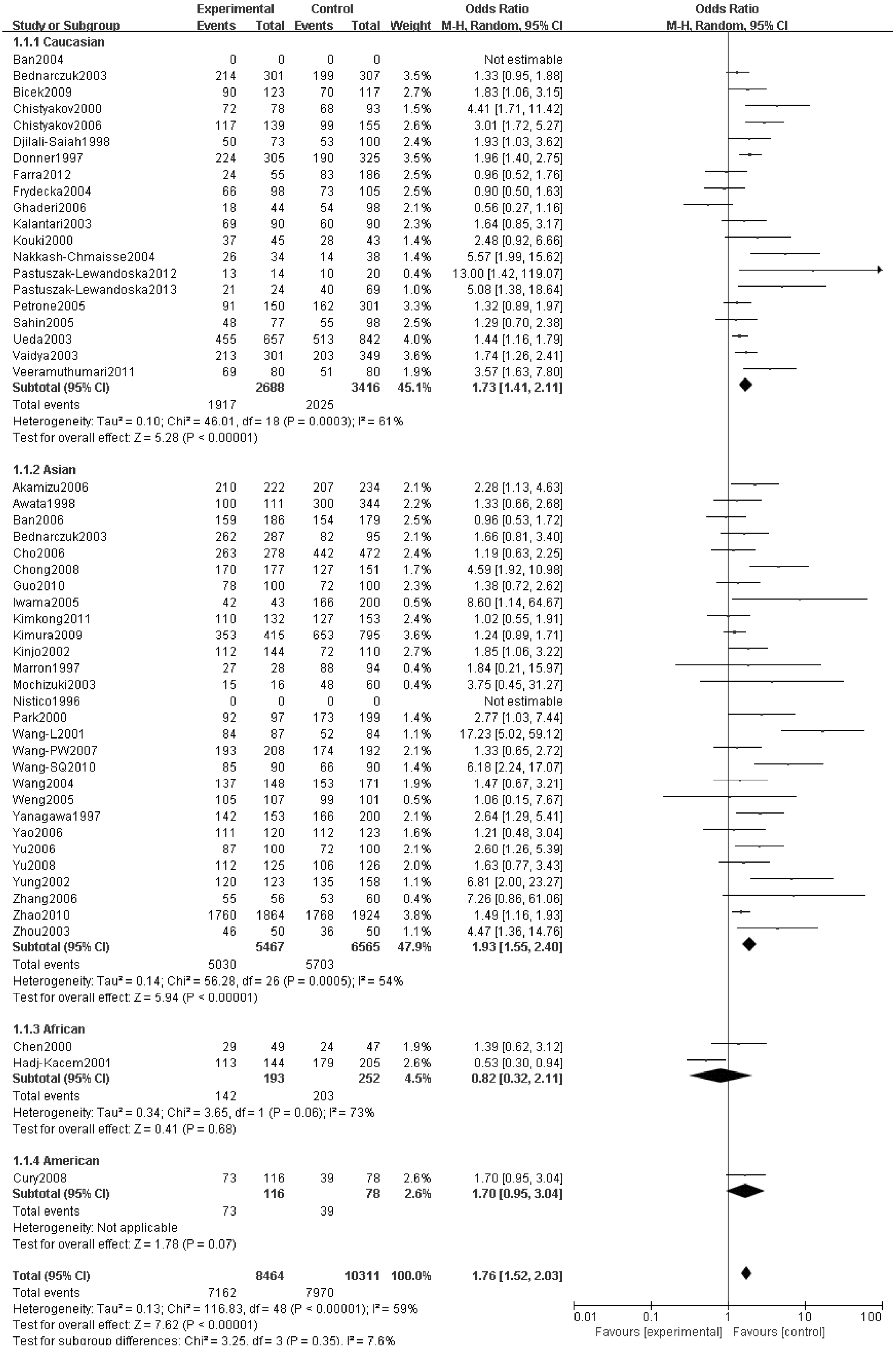

Supplemental Figure 1 Forest plot of the association between a dominant model of A49G polymorphism and GD.

Study or Subgroup	Experimental Events	Experimental Total	Control Events	Control Total	Weight	Odds Ratio M-H, Random, 95% CI
**1.1.1 Caucasian**						
Ban2004	0	0	0	0		Not estimable
Bednarczuk2003	80	301	60	307	2.9%	1.49 [1.02, 2.18]
Bicek2009	17	123	6	117	1.2%	2.97 [1.13, 7.81]
Chistyakov2000	50	78	30	93	2.0%	3.75 [1.99, 7.08]
Chistyakov2006	68	139	39	155	2.5%	2.85 [1.74, 4.66]
Djilali-Saiah1998	13	73	16	100	1.5%	1.14 [0.51, 2.54]
Donner1997	63	305	41	325	2.7%	1.80 [1.17, 2.77]
Farra2012	6	55	16	186	1.1%	1.30 [0.48, 3.50]
Frydecka2004	17	98	20	105	1.7%	0.89 [0.44, 1.82]
Ghaderi2006	3	44	8	98	0.7%	0.82 [0.21, 3.26]
Kalantari2003	20	90	7	90	1.3%	3.39 [1.35, 8.48]
Kouki2000	8	45	5	43	0.8%	1.64 [0.49, 5.49]
Nakkash-Chmaisse2004	3	34	0	38	0.2%	8.56 [0.43, 171.91]
Pastuszak-Lewandoska2012	7	14	5	20	0.6%	3.00 [0.70, 12.88]
Pastuszak-Lewandoska2013	12	24	13	69	1.1%	4.31 [1.58, 11.73]
Petrone2005	23	150	24	301	2.1%	2.09 [1.14, 3.84]
Sahin2005	15	77	7	98	1.2%	3.15 [1.21, 8.16]
Ueda2003	123	657	111	842	3.4%	1.52 [1.15, 2.01]
Vaidya2003	74	301	45	349	2.8%	2.20 [1.46, 3.31]
Veeramuthumari2011	32	80	26	80	1.9%	1.38 [0.72, 2.65]
**Subtotal (95% CI)**		**2688**		**3416**	**31.7%**	**1.93 [1.59, 2.34]**
Total events	634		479			
Heterogeneity: Tau² = 0.05; Chi² = 27.72, df = 18 (P = 0.07); I² = 35%						
Test for overall effect: Z = 6.75 (P < 0.00001)						
**1.1.2 Asian**						
Akamizu2006	112	222	103	234	3.0%	1.29 [0.90, 1.87]
Awata1998	57	111	137	344	2.7%	1.59 [1.04, 2.45]
Ban2006	147	186	69	179	2.6%	6.01 [3.78, 9.56]
Bednarczuk2003	134	287	31	95	2.5%	1.81 [1.11, 2.94]
Cho2006	155	278	245	472	3.3%	1.17 [0.87, 1.57]
Chong2008	97	177	71	151	2.7%	1.37 [0.88, 2.11]
Guo2010	26	102	12	100	1.6%	2.51 [1.19, 5.31]
Iwama2005	17	43	78	200	1.8%	1.02 [0.52, 2.01]
Kimkong2011	61	132	54	153	2.5%	1.58 [0.98, 2.54]
Kimura2009	210	415	295	795	3.5%	1.74 [1.37, 2.21]
Kinjo2002	50	144	26	110	2.2%	1.72 [0.98, 3.00]
Marron1997	16	28	49	94	1.4%	1.22 [0.52, 2.87]
Mochizuki2003	10	16	21	60	0.9%	3.10 [0.99, 9.70]
Nistico1996	0	0	0	0		Not estimable
Park2000	57	97	98	199	2.5%	1.47 [0.90, 2.40]
Wang-L2001	37	87	10	84	1.5%	5.48 [2.50, 12.01]
Wang-PW2007	124	208	97	192	2.9%	1.45 [0.97, 2.15]
Wang-SQ2010	38	90	14	90	1.8%	3.97 [1.96, 8.05]
Wang2004	87	148	81	171	2.7%	1.58 [1.02, 2.47]
Weng2005	78	107	54	101	2.2%	2.34 [1.31, 4.17]
Yanagawa1997	78	153	78	200	2.8%	1.63 [1.06, 2.49]
Yao2006	58	120	55	123	2.4%	1.16 [0.70, 1.92]
Yu2006	51	100	26	100	2.1%	2.96 [1.63, 5.37]
Yu2008	67	125	46	126	2.4%	2.01 [1.21, 3.33]
Yung2002	66	123	76	158	2.6%	1.25 [0.78, 2.00]
Zhang2006	37	56	27	60	1.6%	2.38 [1.12, 5.05]
Zhao2010	1030	1864	945	1924	3.9%	1.28 [1.13, 1.45]
Zhou2003	32	50	16	50	1.4%	3.78 [1.65, 8.65]
**Subtotal (95% CI)**		**5469**		**6565**	**63.7%**	**1.80 [1.54, 2.11]**
Total events	2932		2814			
Heterogeneity: Tau² = 0.10; Chi² = 84.56, df = 26 (P < 0.00001); I² = 69%						
Test for overall effect: Z = 7.35 (P < 0.00001)						
**1.1.3 African**						
Chen2000	4	49	5	47	0.7%	0.75 [0.19, 2.97]
Hadj-Kacem2001	50	144	85	205	2.7%	0.75 [0.48, 1.17]
**Subtotal (95% CI)**		**193**		**252**	**3.4%**	**0.75 [0.49, 1.14]**
Total events	54		90			
Heterogeneity: Tau² = 0.00; Chi² = 0.00, df = 1 (P = 0.99); I² = 0%						
Test for overall effect: Z = 1.34 (P = 0.18)						
**1.1.4 American**						
Cury2008	15	116	7	78	1.2%	1.51 [0.58, 3.88]
**Subtotal (95% CI)**		**116**		**78**	**1.2%**	**1.51 [0.58, 3.88]**
Total events	15		7			
Heterogeneity: Not applicable						
Test for overall effect: Z = 0.85 (P = 0.40)						
**Total (95% CI)**		**8466**		**10311**	**100.0%**	**1.79 [1.58, 2.02]**
Total events	3635		3390			
Heterogeneity: Tau² = 0.10; Chi² = 129.67, df = 48 (P < 0.00001); I² = 63%						
Test for overall effect: Z = 9.18 (P < 0.00001)						
Test for subgroup differences: Chi² = 16.73, df = 3 (P = 0.0008), I² = 82.1%						

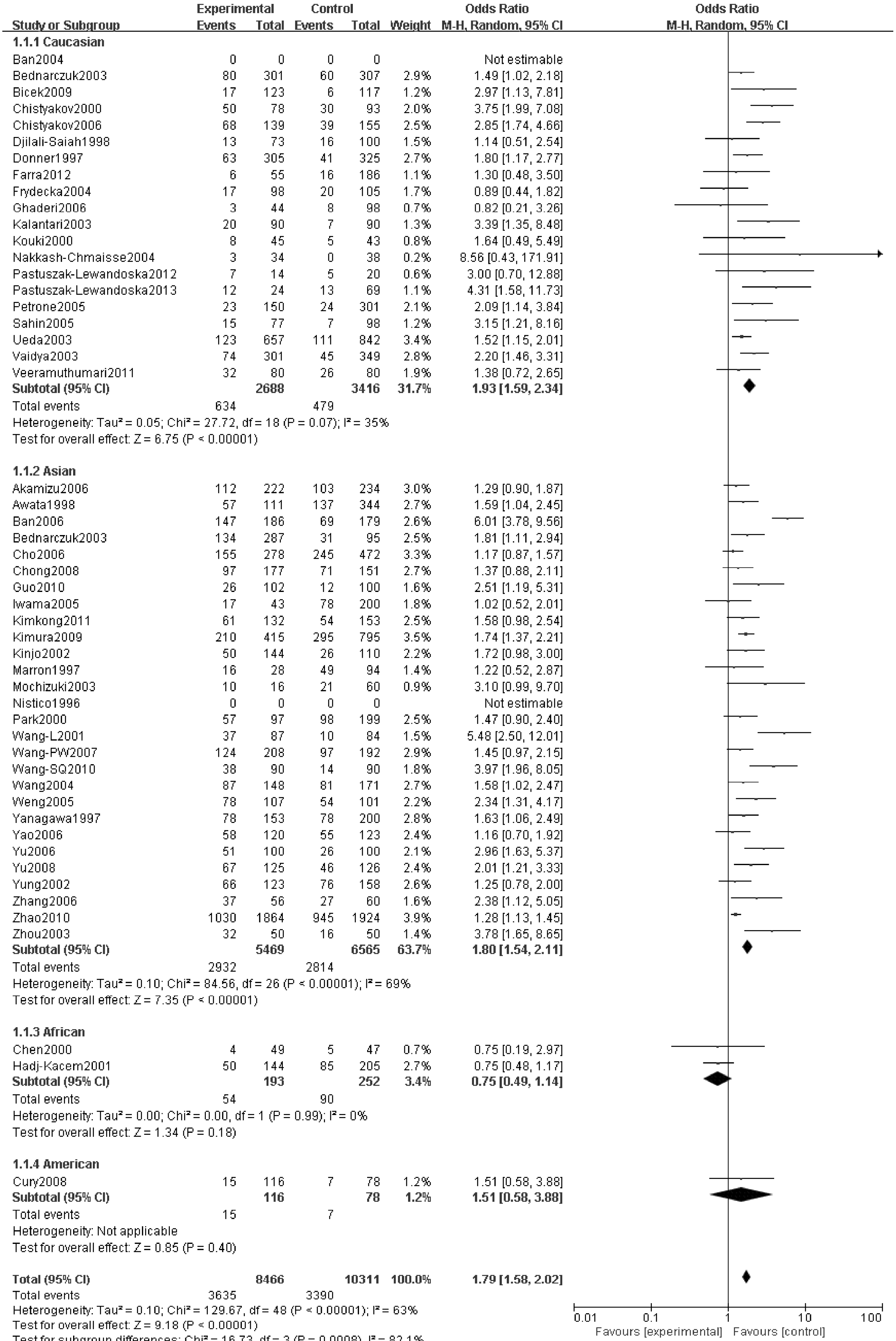

Supplemental Figure 2 Forest plot of the association between a recessive model of A49G polymorphism and GD.

Study or Subgroup	Experimental Events	Experimental Total	Control Events	Control Total	Weight	Odds Ratio M-H, Fixed, 95% CI
**1.1.1 Caucasian**						
Bicek2009	15	66	6	53	3.1%	2.30 [0.83, 6.43]
Dallos2008	13	29	31	139	3.5%	2.83 [1.23, 6.52]
Donner1997	16	34	68	251	5.1%	2.39 [1.15, 4.96]
Farra2012	6	42	16	119	4.3%	1.07 [0.39, 2.95]
Ghaderi2006	6	25	8	52	2.4%	1.74 [0.53, 5.69]
Kouki2000	3	10	5	20	1.4%	1.29 [0.24, 6.96]
Kucharska2009	31	60	15	37	5.3%	1.57 [0.68, 3.59]
Pastuszak-Lewandoska2012	6	9	5	15	0.7%	4.00 [0.69, 23.09]
Pastuszak-Lewandoska2013	14	17	13	42	0.8%	10.41 [2.55, 42.56]
Petrone2001	16	74	25	163	7.3%	1.52 [0.76, 3.06]
Sahin2009	21	106	7	50	4.5%	1.52 [0.60, 3.85]
Ueda2003	33	91	111	440	14.5%	1.69 [1.05, 2.72]
**Subtotal (95% CI)**		**563**		**1381**	**52.9%**	**1.92 [1.50, 2.46]**
Total events	180		310			
Heterogeneity: Chi² = 10.21, df = 11 (P = 0.51); I² = 0%						
Test for overall effect: Z = 5.23 (P < 0.00001)						
**1.1.2 Asian**						
Akamizu2006	69	78	103	130	5.3%	2.01 [0.89, 4.53]
Awata1998	45	51	170	228	4.4%	2.56 [1.04, 6.31]
Ban2005	77	84	95	109	4.1%	1.62 [0.62, 4.22]
Ban2006	74	91	69	94	7.6%	1.58 [0.78, 3.17]
Park2000	45	58	98	124	8.3%	0.92 [0.43, 1.95]
Terauchi2003	30	33	36	57	1.4%	5.83 [1.58, 21.47]
Tomoyose2002	62	74	62	112	4.8%	4.17 [2.02, 8.57]
Ying2012	46	97	17	66	6.3%	2.60 [1.32, 5.13]
Yu2008	41	46	46	66	2.4%	3.57 [1.23, 10.36]
Zhou2003	24	32	16	30	2.5%	2.63 [0.90, 7.69]
**Subtotal (95% CI)**		**644**		**1016**	**47.1%**	**2.29 [1.76, 2.98]**
Total events	513		712			
Heterogeneity: Chi² = 12.88, df = 9 (P = 0.17); I² = 30%						
Test for overall effect: Z = 6.18 (P < 0.00001)						
**Total (95% CI)**		**1207**		**2397**	**100.0%**	**2.10 [1.75, 2.51]**
Total events	693		1022			
Heterogeneity: Chi² = 23.86, df = 21 (P = 0.30); I² = 12%						
Test for overall effect: Z = 8.11 (P < 0.00001)						
Test for subgroup differences: Chi² = 0.91, df = 1 (P = 0.34), I² = 0%						

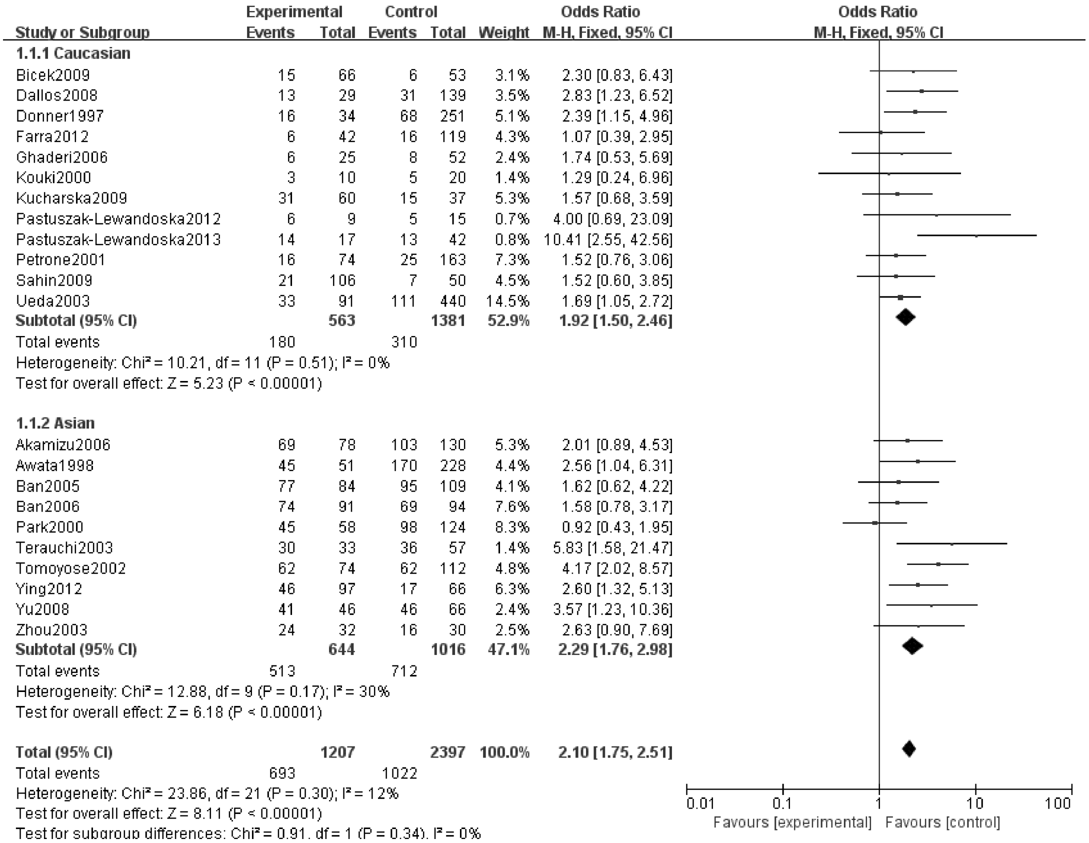

Supplemental Figure 3 Forest plot of the association between an additive model of A49G polymorphism and HT.

Study or Subgroup	Experimental Events	Experimental Total	Control Events	Control Total	Weight	Odds Ratio M-H, Random, 95% CI
**1.1.1 Caucasian**						
Bicek2009	61	112	70	117	6.0%	0.80 [0.48, 1.36]
Dallos2008	47	63	122	230	5.2%	2.60 [1.39, 4.85]
Donner1997	55	73	283	466	5.7%	1.98 [1.12, 3.47]
Farra2012	37	73	83	186	5.8%	1.28 [0.74, 2.19]
Ghaderi2006	18	37	54	98	4.4%	0.77 [0.36, 1.65]
Kouki2000	11	18	28	43	2.7%	0.84 [0.27, 2.62]
Kucharska2009	71	100	79	101	5.1%	0.68 [0.36, 1.29]
Pastuszak-Lewandoska2012	25	28	10	20	1.8%	8.33 [1.89, 36.76]
Pastuszak-Lewandoska2013	22	25	40	69	2.2%	5.32 [1.45, 19.46]
Petrone2001	68	126	163	301	6.8%	0.99 [0.65, 1.51]
Sahin2009	112	197	55	98	6.2%	1.03 [0.63, 1.68]
Ueda2003	152	210	513	842	7.5%	1.68 [1.21, 2.34]
**Subtotal (95% CI)**		**1062**		**2571**	**59.3%**	**1.34 [0.99, 1.80]**
Total events	679		1500			
Heterogeneity: Tau² = 0.16; Chi² = 31.79, df = 11 (P = 0.0008); I² = 65%						
Test for overall effect: Z = 1.88 (P = 0.06)						
**1.1.2 Asian**						
Akamizu2006	130	139	207	234	4.2%	1.88 [0.86, 4.13]
Awata1998	82	88	367	425	3.7%	2.16 [0.90, 5.18]
Ban2005	126	133	165	179	3.4%	1.53 [0.60, 3.90]
Ban2006	166	183	154	179	5.0%	1.59 [0.82, 3.05]
Park2000	97	110	133	159	4.6%	1.46 [0.71, 2.98]
Terauchi2003	67	70	84	105	2.3%	5.58 [1.60, 19.52]
Tomoyose2002	131	143	149	199	4.9%	3.66 [1.87, 7.18]
Ying2012	99	150	71	120	6.2%	1.34 [0.82, 2.20]
Yu2008	75	80	106	126	3.1%	2.83 [1.02, 7.88]
Zhou2003	38	46	36	50	3.2%	1.85 [0.69, 4.93]
**Subtotal (95% CI)**		**1142**		**1776**	**40.7%**	**1.93 [1.49, 2.51]**
Total events	1011		1472			
Heterogeneity: Tau² = 0.02; Chi² = 10.19, df = 9 (P = 0.34); I² = 12%						
Test for overall effect: Z = 4.96 (P < 0.00001)						
**Total (95% CI)**		**2204**		**4347**	**100.0%**	**1.57 [1.26, 1.96]**
Total events	1690		2972			
Heterogeneity: Tau² = 0.14; Chi² = 48.70, df = 21 (P = 0.0006); I² = 57%						
Test for overall effect: Z = 4.01 (P < 0.0001)						
Test for subgroup differences: Chi² = 3.31, df = 1 (P = 0.07), I² = 69.8%						

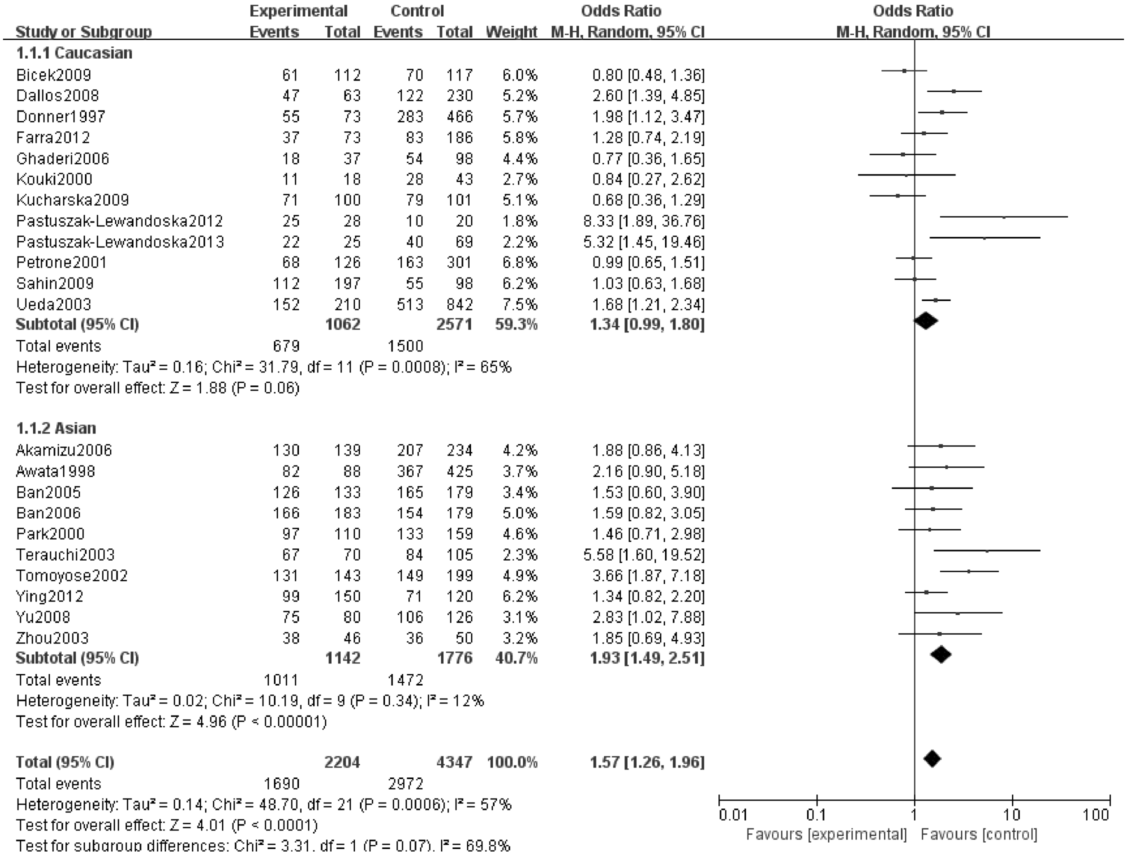

Supplemental Figure 4 Forest plot of the association between a dominant model of A49G polymorphism and HT.

Study or Subgroup	Experimental Events	Experimental Total	Control Events	Control Total	Weight	Odds Ratio M-H, Random, 95% CI
**1.1.1 Caucasian**						
Bicek2009	15	112	6	117	3.1%	2.86 [1.07, 7.66]
Dallos2008	13	63	31	230	4.5%	1.67 [0.81, 3.42]
Donner1997	16	73	68	466	5.2%	1.64 [0.89, 3.03]
Farra2012	6	73	16	186	3.1%	0.95 [0.36, 2.54]
Ghaderi2006	6	37	8	98	2.5%	2.18 [0.70, 6.77]
Kouki2000	3	18	5	43	1.5%	1.52 [0.32, 7.17]
Kucharska2009	31	100	15	101	4.7%	2.58 [1.29, 5.15]
Pastuszak-Lewandoska2012	6	28	5	20	1.9%	0.82 [0.21, 3.18]
Pastuszak-Lewandoska2013	14	25	13	69	3.0%	5.48 [2.03, 14.81]
Petrone2001	16	126	25	301	4.8%	1.61 [0.83, 3.12]
Sahin2009	21	197	7	0		Not estimable
Ueda2003	33	210	111	842	6.8%	1.23 [0.81, 1.87]
**Subtotal (95% CI)**		**1062**		**2473**	**41.2%**	**1.73 [1.32, 2.27]**
Total events	180		310			
Heterogeneity: Tau² = 0.04; Chi² = 12.76, df = 10 (P = 0.24); I² = 22%						
Test for overall effect: Z = 3.96 (P < 0.0001)						
**1.1.2 Asian**						
Akamizu2006	69	139	103	234	6.9%	1.25 [0.82, 1.91]
Awata1998	45	88	170	425	6.5%	1.57 [0.99, 2.49]
Ban2005	77	133	95	179	6.6%	1.22 [0.77, 1.91]
Ban2006	74	183	69	179	6.8%	1.08 [0.71, 1.65]
Park2000	45	110	98	159	6.2%	0.43 [0.26, 0.71]
Terauchi2003	30	70	36	105	5.2%	1.44 [0.77, 2.68]
Tomoyose2002	62	143	62	199	6.6%	1.69 [1.08, 2.64]
Ying2012	46	150	17	120	5.2%	2.68 [1.44, 4.98]
Yu2008	41	80	46	126	5.6%	1.83 [1.04, 3.23]
Zhou2003	24	46	16	30	3.3%	0.95 [0.38, 2.40]
**Subtotal (95% CI)**		**1142**		**1756**	**58.8%**	**1.29 [0.96, 1.72]**
Total events	513		712			
Heterogeneity: Tau² = 0.15; Chi² = 28.86, df = 9 (P = 0.0007); I² = 69%						
Test for overall effect: Z = 1.69 (P = 0.09)						
**Total (95% CI)**		**2204**		**4229**	**100.0%**	**1.46 [1.19, 1.81]**
Total events	693		1022			
Heterogeneity: Tau² = 0.12; Chi² = 45.40, df = 20 (P = 0.0010); I² = 56%						
Test for overall effect: Z = 3.57 (P = 0.0004)						
Test for subgroup differences: Chi² = 2.12, df = 1 (P = 0.15), I² = 52.8%						

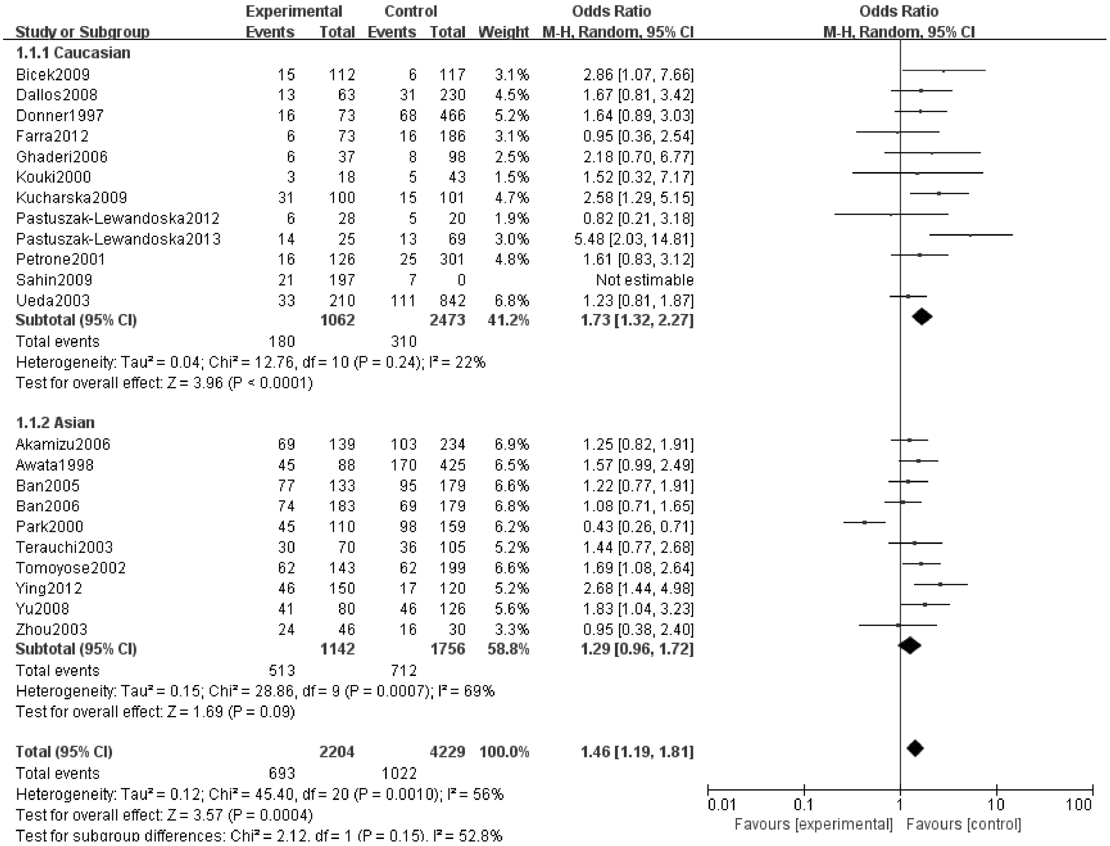

Supplemental Figure 5 Forest plot of the association between a recessive model of A49G polymorphism and HT.

Study or Subgroup	Experimental Events	Experimental Total	Control Events	Control Total	Weight	Odds Ratio M-H, Fixed, 95% CI
**1.1.1 Caucasian**						
Ban2004	0	0	0	0		Not estimable
Bednarczuk 2006	140	167	110	152	6.0%	1.98 [1.15, 3.41]
Bicek2009	50	66	30	56	2.5%	2.71 [1.25, 5.85]
Chistiakov 2006	61	87	33	96	3.0%	4.48 [2.40, 8.35]
Frydecka 2006	37	47	41	59	2.5%	1.62 [0.67, 3.96]
Ghaderi2006	7	22	20	49	2.7%	0.68 [0.23, 1.96]
Petrone2005	45	71	69	152	5.2%	2.08 [1.17, 3.71]
Ueda2003	267	357	240	428	17.6%	2.32 [1.71, 3.16]
**Subtotal (95% CI)**		**817**		**992**	**39.5%**	**2.27 [1.85, 2.79]**
Total events	607		543			
Heterogeneity: Chi² = 10.65, df = 6 (P = 0.10); I² = 44%						
Test for overall effect: Z = 7.85 (P < 0.00001)						
**1.1.2 Asian**						
Akamizu2006	140	145	113	128	1.3%	3.72 [1.31, 10.54]
Ban 2006	198	206	95	109	1.5%	3.65 [1.48, 8.99]
Ban2005	91	93	95	109	0.6%	6.71 [1.48, 30.33]
Cho2006	194	201	334	347	2.7%	1.08 [0.42, 2.75]
Chong2008	125	129	88	100	1.0%	4.26 [1.33, 13.65]
Hiromatsu 2006	180	191	45	55	1.3%	3.64 [1.45, 9.09]
Ikegami2006	130	142	395	432	5.3%	1.01 [0.51, 2.00]
Kimkong2011	78	86	82	94	2.3%	1.43 [0.55, 3.68]
Kimura2009	267	288	735	819	8.9%	1.45 [0.88, 2.39]
Qu2014	1989	2103	1550	1686	29.9%	1.53 [1.18, 1.98]
Tai2008	136	141	372	404	2.2%	2.34 [0.89, 6.13]
Wang 2006	106	112	81	99	1.5%	3.93 [1.49, 10.34]
Wang-PW2007	138	143	125	134	1.4%	1.99 [0.65, 6.09]
Weng2005	78	80	54	56	0.5%	1.44 [0.20, 10.57]
**Subtotal (95% CI)**		**4060**		**4572**	**60.5%**	**1.79 [1.50, 2.14]**
Total events	3850		4164			
Heterogeneity: Chi² = 20.66, df = 13 (P = 0.08); I² = 37%						
Test for overall effect: Z = 6.42 (P < 0.00001)						
**Total (95% CI)**		**4877**		**5564**	**100.0%**	**1.98 [1.73, 2.27]**
Total events	4457		4707			
Heterogeneity: Chi² = 34.68, df = 20 (P = 0.02); I² = 42%						
Test for overall effect: Z = 9.96 (P < 0.00001)						
Test for subgroup differences: Chi² = 2.96, df = 1 (P = 0.09), I² = 66.3%						

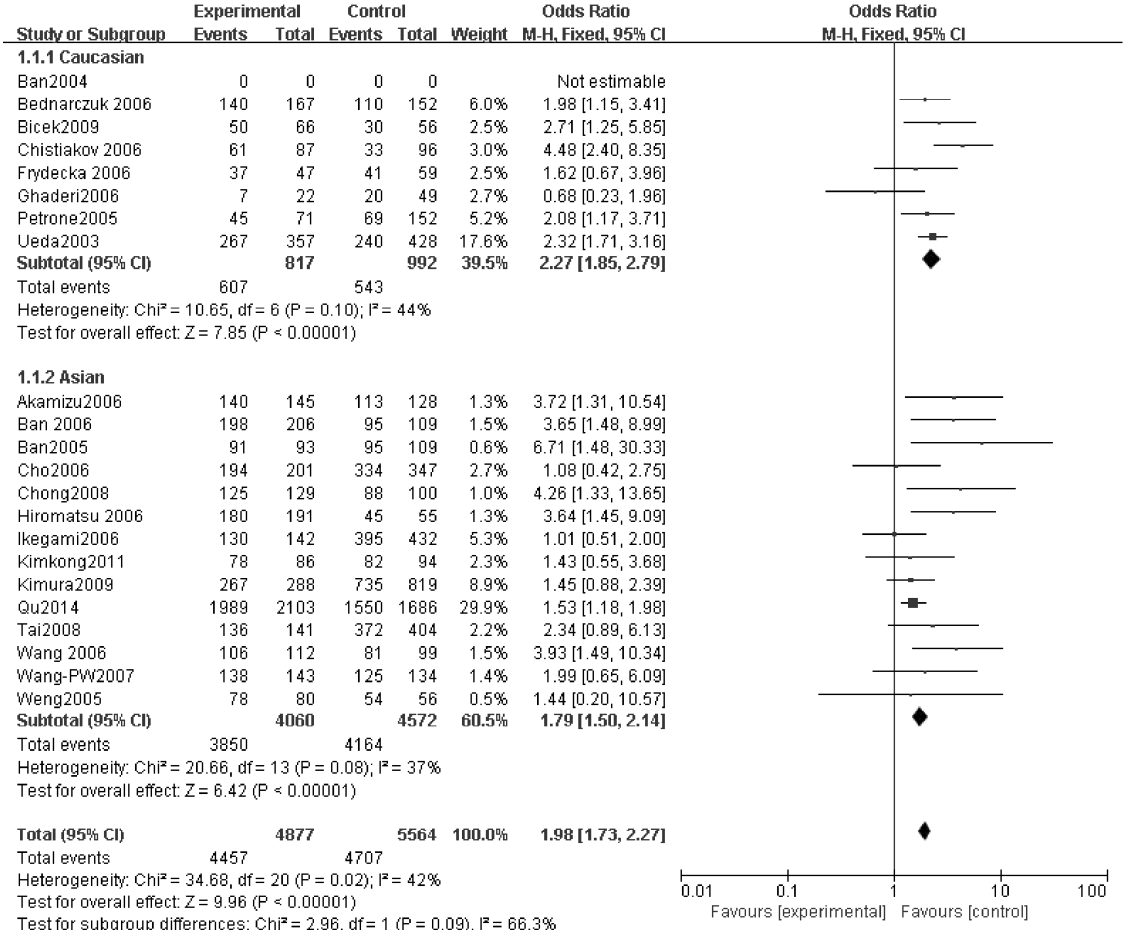

Supplemental Figure 6 Forest plot of the association between an additive model of CT60 polymorphism and GD.

Study or Subgroup	Experimental Events	Experimental Total	Control Events	Control Total	Weight	Odds Ratio M-H, Fixed, 95% CI
**1.1.1 Caucasian**						
Ban2004	0	0	0	0		Not estimable
Bednarczuk 2006	257	284	256	298	6.3%	1.56 [0.93, 2.61]
Bicek2009	107	123	91	117	3.2%	1.91 [0.97, 3.78]
Chistiakov 2006	113	139	92	155	4.3%	2.98 [1.75, 5.07]
Frydecka 2006	88	98	80	98	2.2%	1.98 [0.86, 4.54]
Ghaderi2006	28	43	70	99	3.9%	0.77 [0.36, 1.66]
Petrone2005	124	150	218	301	6.6%	1.82 [1.11, 2.97]
Ueda2003	568	658	635	823	20.4%	1.87 [1.42, 2.46]
**Subtotal (95% CI)**		**1495**		**1891**	**46.8%**	**1.84 [1.53, 2.21]**
Total events	1285		1442			
Heterogeneity: Chi² = 8.54, df = 6 (P = 0.20); I² = 30%						
Test for overall effect: Z = 6.55 (P < 0.00001)						
**1.1.2 Asian**						
Akamizu2006	210	215	192	207	1.2%	3.28 [1.17, 9.20]
Ban 2006	294	302	165	179	1.4%	3.12 [1.28, 7.59]
Ban2005	129	131	165	179	0.6%	5.47 [1.22, 24.51]
Cho2006	271	278	459	472	2.3%	1.10 [0.43, 2.78]
Chong2008	173	177	139	151	0.9%	3.73 [1.18, 11.83]
Hiromatsu 2006	276	287	85	95	1.3%	2.95 [1.21, 7.19]
Ikegami2006	201	213	678	715	4.6%	0.91 [0.47, 1.79]
Kimkong2011	124	132	141	153	2.1%	1.32 [0.52, 3.33]
Kimura2009	394	415	1251	1335	7.9%	1.26 [0.77, 2.06]
Qu2014	2476	2590	2024	2160	25.6%	1.46 [1.13, 1.88]
Tai2008	184	189	588	620	1.9%	2.00 [0.77, 5.21]
Wang 2006	136	142	153	171	1.5%	2.67 [1.03, 6.91]
Wang-PW2007	184	189	183	192	1.3%	1.81 [0.60, 5.50]
Weng2005	105	107	99	101	0.5%	1.06 [0.15, 7.67]
**Subtotal (95% CI)**		**5367**		**6730**	**53.2%**	**1.62 [1.36, 1.94]**
Total events	5157		6322			
Heterogeneity: Chi² = 16.95, df = 13 (P = 0.20); I² = 23%						
Test for overall effect: Z = 5.39 (P < 0.00001)						
**Total (95% CI)**		**6862**		**8621**	**100.0%**	**1.72 [1.52, 1.96]**
Total events	6442		7764			
Heterogeneity: Chi² = 26.60, df = 20 (P = 0.15); I² = 25%						
Test for overall effect: Z = 8.42 (P < 0.00001)						
Test for subgroup differences: Chi² = 0.92, df = 1 (P = 0.34), I² = 0%						

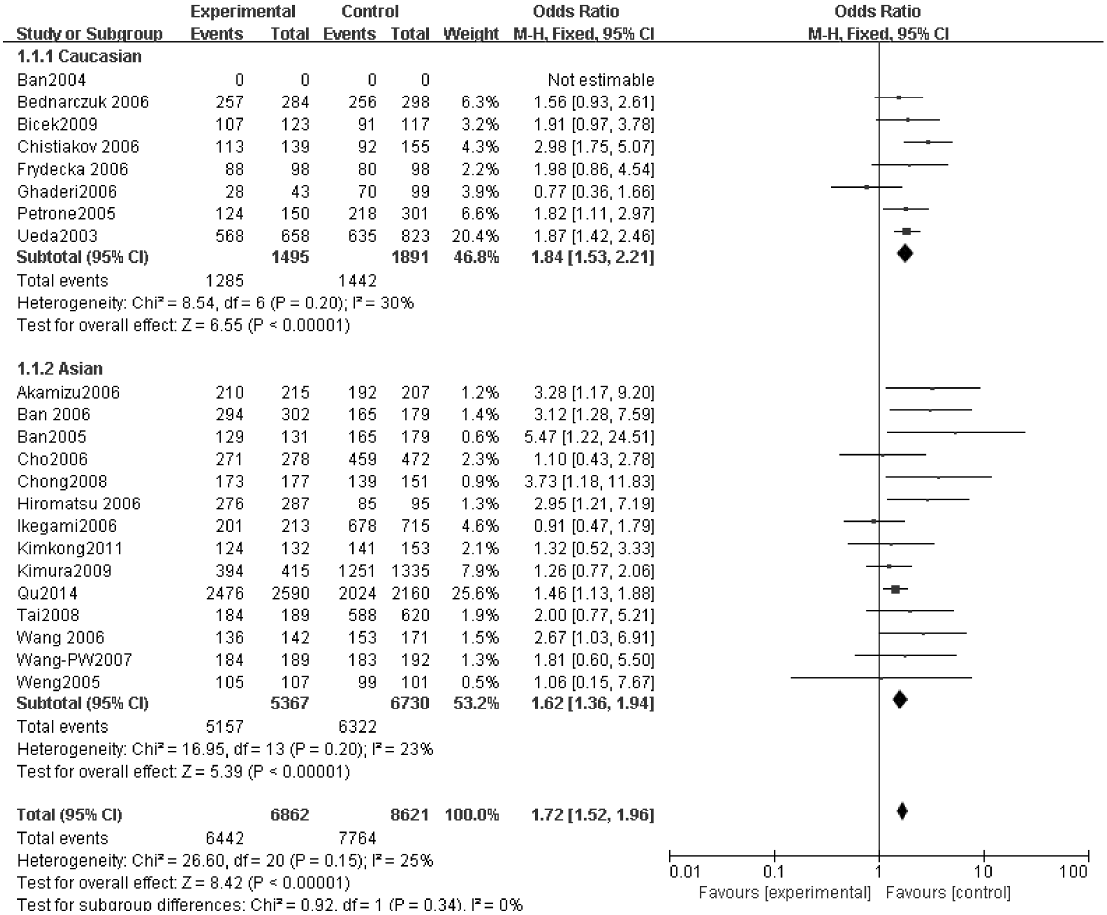

Supplemental Figure 7 Forest plot of the association between a dominant model of CT60 polymorphism and GD.

Study or Subgroup	Experimental Events	Experimental Total	Control Events	Control Total	Weight	Odds Ratio M-H, Random, 95% CI
**1.1.1 Caucasian**						
Ban2004	0	0	0	0		Not estimable
Bednarczuk 2006	140	284	110	298	5.7%	1.66 [1.19, 2.31]
Bicek2009	50	123	30	117	3.2%	1.99 [1.15, 3.44]
Chistiakov 2006	61	139	33	155	3.6%	2.89 [1.74, 4.81]
Frydecka 2006	37	98	41	98	3.1%	0.84 [0.48, 1.50]
Ghaderi2006	7	43	20	99	1.4%	0.77 [0.30, 1.98]
Petrone2005	45	150	69	301	4.3%	1.44 [0.93, 2.24]
Ueda2003	267	658	240	823	7.7%	1.66 [1.34, 2.06]
**Subtotal (95% CI)**		**1495**		**1891**	**29.0%**	**1.58 [1.24, 2.03]**
Total events	607		543			
Heterogeneity: Tau² = 0.05; Chi² = 13.20, df = 6 (P = 0.04); I² = 55%						
Test for overall effect: Z = 3.63 (P = 0.0003)						
**1.1.2 Asian**						
Akamizu2006	140	215	113	207	4.9%	1.55 [1.05, 2.30]
Ban 2006	198	302	95	179	5.1%	1.68 [1.15, 2.46]
Ban2005	91	131	95	179	3.9%	2.01 [1.25, 3.23]
Cho2006	194	278	334	472	5.9%	0.95 [0.69, 1.32]
Chong2008	125	177	88	151	4.1%	1.72 [1.09, 2.72]
Hiromatsu 2006	180	287	45	95	4.0%	1.87 [1.17, 2.99]
Ikegami2006	130	213	395	715	6.0%	1.27 [0.93, 1.73]
Kimkong2011	78	132	82	153	4.0%	1.25 [0.78, 2.00]
Kimura2009	267	415	735	1335	7.5%	1.47 [1.17, 1.85]
Qu2014	1989	2590	1550	2160	9.2%	1.30 [1.14, 1.48]
Tai2008	136	189	372	620	5.4%	1.71 [1.20, 2.44]
Wang 2006	106	142	81	171	3.8%	3.27 [2.02, 5.30]
Wang-PW2007	138	189	125	192	4.3%	1.45 [0.94, 2.25]
Weng2005	78	107	54	101	3.0%	2.34 [1.31, 4.17]
**Subtotal (95% CI)**		**5367**		**6730**	**71.0%**	**1.55 [1.35, 1.78]**
Total events	3850		4164			
Heterogeneity: Tau² = 0.03; Chi² = 28.68, df = 13 (P = 0.007); I² = 55%						
Test for overall effect: Z = 6.17 (P < 0.00001)						
**Total (95% CI)**		**6862**		**8621**	**100.0%**	**1.56 [1.39, 1.76]**
Total events	4457		4707			
Heterogeneity: Tau² = 0.04; Chi² = 43.89, df = 20 (P = 0.002); I² = 54%						
Test for overall effect: Z = 7.34 (P < 0.00001)						
Test for subgroup differences: Chi² = 0.02, df = 1 (P = 0.88), I² = 0%						

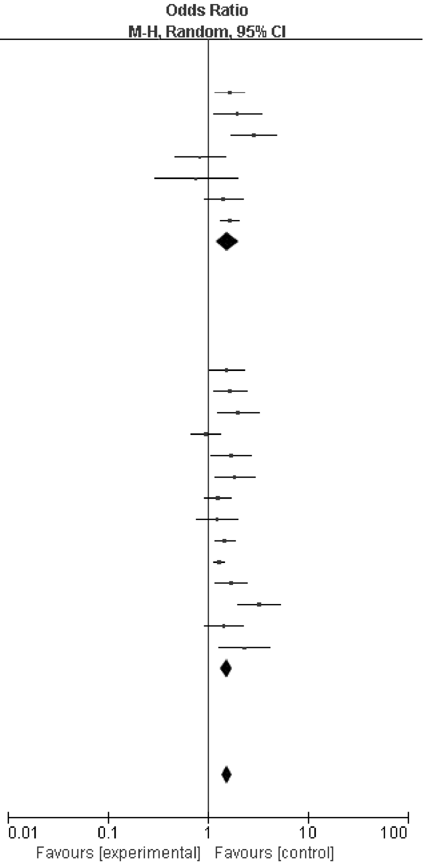

Supplemental Figure 8 Forest plot of the association between a recessive model of CT60 polymorphism and GD.

Study or Subgroup	Experimental Events	Experimental Total	Control Events	Control Total	Weight	Odds Ratio M-H, Random, 95% CI
**1.1.1 Caucasian**						
Bicek2009	37	60	30	56	13.6%	1.39 [0.67, 2.92]
Dallos2008	31	35	20	45	10.7%	9.69 [2.93, 32.03]
Ghaderi2006	8	19	20	49	11.4%	1.05 [0.36, 3.09]
Ueda2003	85	115	111	440	15.1%	8.40 [5.26, 13.42]
**Subtotal (95% CI)**		**229**		**590**	**50.8%**	**3.33 [1.04, 10.67]**
Total events	161		181			
Heterogeneity: Tau² = 1.21; Chi² = 25.05, df = 3 (P < 0.0001); I² = 88%						
Test for overall effect: Z = 2.02 (P = 0.04)						
**1.1.2 Asian**						
Akamizu2006	84	91	103	130	12.7%	3.15 [1.30, 7.58]
Ban 2006	103	113	95	109	12.8%	1.52 [0.64, 3.58]
Ban2005	77	84	95	109	12.2%	1.62 [0.62, 4.22]
Ikegami2006	83	87	395	432	11.5%	1.94 [0.67, 5.60]
**Subtotal (95% CI)**		**375**		**780**	**49.2%**	**1.98 [1.24, 3.15]**
Total events	347		688			
Heterogeneity: Tau² = 0.00; Chi² = 1.61, df = 3 (P = 0.66); I² = 0%						
Test for overall effect: Z = 2.88 (P = 0.004)						
**Total (95% CI)**		**604**		**1370**	**100.0%**	**2.58 [1.33, 5.01]**
Total events	508		869			
Heterogeneity: Tau² = 0.70; Chi² = 34.17, df = 7 (P < 0.0001); I² = 80%						
Test for overall effect: Z = 2.81 (P = 0.005)						
Test for subgroup differences: Chi² = 0.66, df = 1 (P = 0.42), I² = 0%						

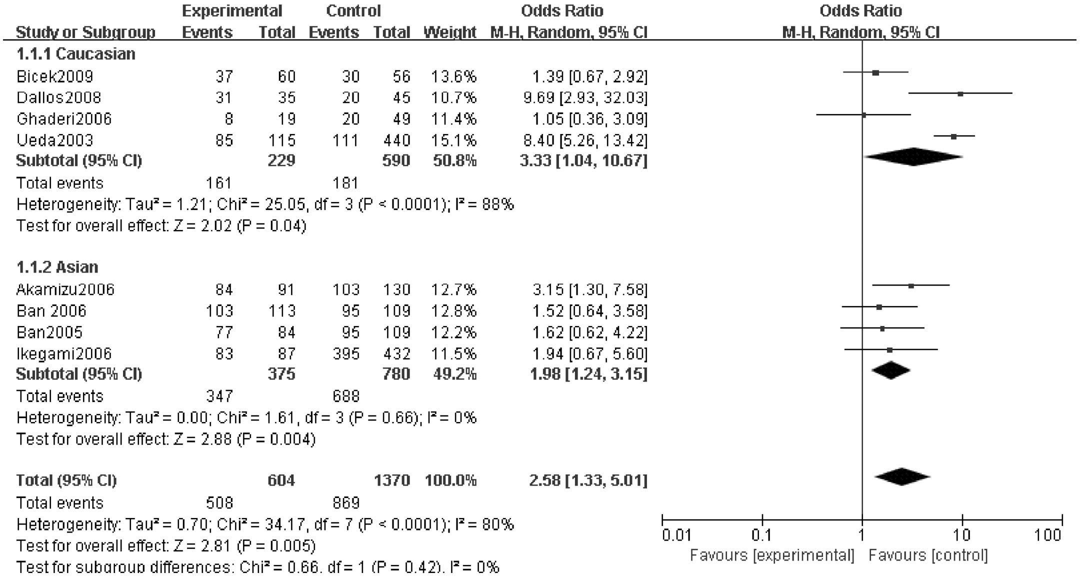

Supplemental Figure 9 Forest plot of the association between an additive model of CT60 polymorphism and HT.

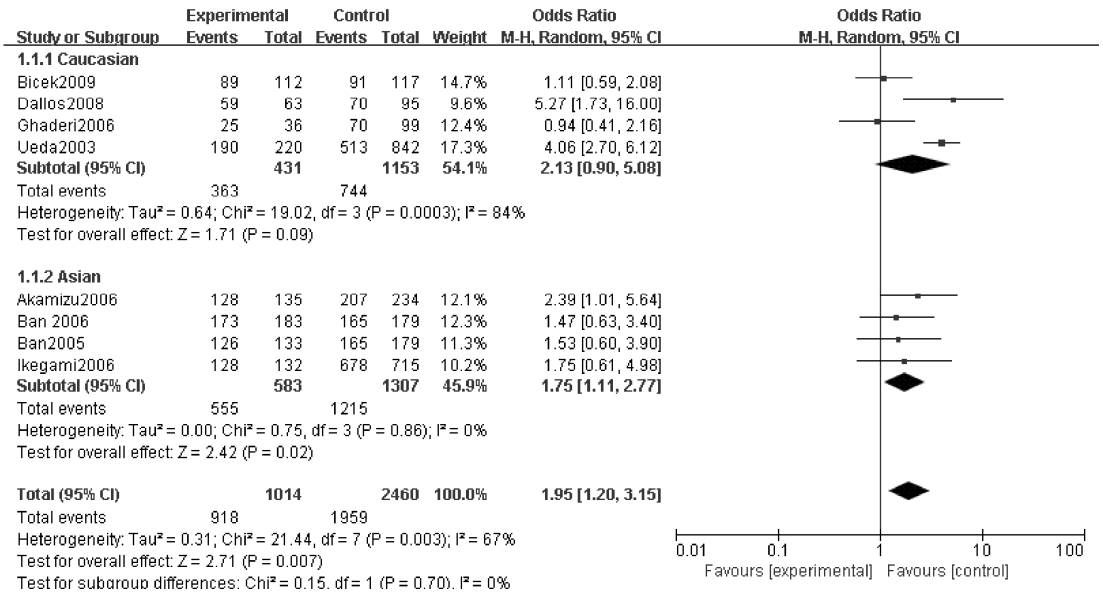

Supplemental Figure 10 Forest plot of the association between a dominant model of CT60 polymorphism and HT.

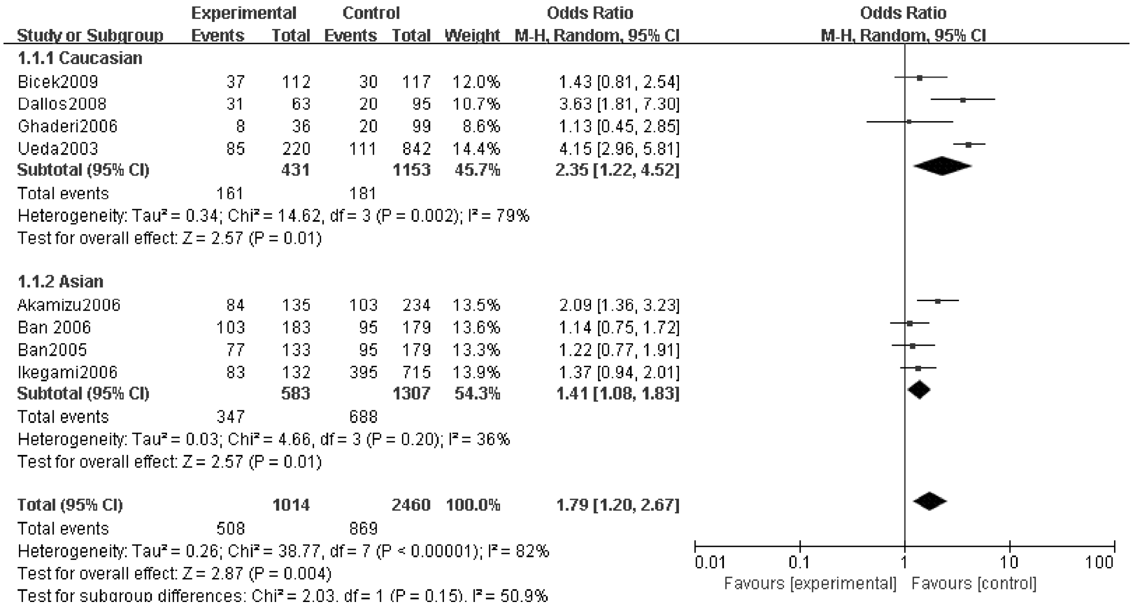

Supplemental Figure 11 Forest plot of the association between a recessive model of CT60 polymorphism and HT.
